# Eyes on privacy: acceptance of video-based AAL impacted by activities being filmed

**DOI:** 10.3389/fpubh.2023.1186944

**Published:** 2023-07-04

**Authors:** Caterina Maidhof, Julia Offermann, Martina Ziefle

**Affiliations:** Human-Computer Interaction Center, Chair of Communication Science, RWTH Aachen University, Aachen, Germany

**Keywords:** video-based ambient and assisted living (AAL), assistive technology, cameras, technology acceptance, privacy need, privacy perception, activities of daily living (ADLs)

## Abstract

**Introduction:**

The use of video-based ambient assisted living (AAL) technologies represents an innovative approach to supporting older adults living as independently and autonomously as possible in their homes. These visual devices have the potential to increase security, perceived safety, and relief for families and caregivers by detecting, among others, emergencies or serious health situations. Despite these potentials and advantages, using video-based technologies for monitoring different activities in everyday life evokes concerns about privacy intrusion and data security. For a sustainable design and adoption of such technical innovations, a detailed analysis of future users' acceptance, including perceived benefits and barriers is required and possible effects and privacy needs of different activities being filmed should be taken into account.

**Methods:**

Therefore, the present study investigated the acceptance and benefit-barrier-perception of using video-based AAL technologies for different activities of daily living based on a scenario-based online survey (*N* = 146).

**Results:**

In the first step, the results identified distinct evaluation patterns for 25 activities of daily living with very high (e.g., changing clothes, showering) and very low privacy needs (e.g., gardening, eating, and drinking). In a second step, three exemplary activity types were compared regarding acceptance, perceived benefits, and barriers. The acceptance and the perceived benefits of using video-based AAL technologies revealed to be higher in household and social activities compared to intimate activities. The strongest barrier perception was found for intimate activities and mainly regarded privacy concerns.

**Discussion:**

The results can be used to derive design and information recommendations for the conception, development, and communication of video-based AAL technologies in order to meet the requirements and needs of future users.

## 1. Introduction

Older adults' desire to age independently in their own home may increasingly be reached with the support of ambient-assisted living (AAL) technologies (1–5). For this support, a variety of wearable and ambient sensors are widely applicable and combinable to be installed in the own home (6, 7). AAL technologies have the potential to help people in need of care through, for instance, the monitoring of activity and behavior change, the detection of emergencies (e.g., falls), the stimulation of cognitive functions, the monitoring of physiological parameters, or through the provision of communication services (1, 8). The overall intention of AAL is to strengthen the autonomy and independence of people in need of help at home, to lower the care burden of caregivers and to improve the quality of life for all parties involved (1, 2, 9). Recent advancements in computer vision made video-based technology an attractive option for AAL usage (10). In fact, being similar to human vision, humans can easily interpret data captured through visual sensors. In addition, high-quality interpretation of the sensory output is possible with data coming from just one single visual sensor. This sensor monitors an entire room and makes it unnecessary to install other sensors (6, 10). From a technical and economic perspective, these developments might be advantageous. However, studies dealing with the acceptance of video-based AAL technologies, which are still outnumbered, show that the use of video-based AAL is critical from an acceptance point of view and bears considerable privacy costs (11–13). Thus, despite being highly positive from a technological and economic point of view, video-based AAL technology provokes sensitive social issues that need in-depth discussion and research on acceptance boundaries (14, 15).

Traditionally, technology acceptance is measured with the Technology Acceptance Model (TAM) or its extension, the Unified Theory of Acceptance (UTAUT) (16–18). The core variables of these established models are the perceived ease of use (PEOU) and perceived usefulness (PU) of a technology. The models demonstrated to explain 40–70% of an individual's behavioral intention to use technology in several contexts including healthcare (18). Behavioral intentions are specific attitudes toward performing behaviors regarding an object or target, in this case, video-based AAL technology (19).

In the context of (video-based) AAL technology, the behavioral intention to use such technology in the own home is often determined by a weighing-off of benefits and barriers (3, 12, 14, 20, 21). The weighing regards positive aspects of AAL technology such as increased independence, increased safety, or perceived usefulness (14, 15, 22) which are contrasted and traded-off with the perceived barriers. Among the negative aspects, are a lack of control over technology, false alarms, obtrusiveness, high costs but also privacy concerns such as fear of data misuse, unauthorized data access, and ongoing surveillance (23–25). Privacy issues are especially high when visual sensors in the own home are involved in AAL monitoring (11, 13, 26, 27). Indeed, potential users oftentimes mention the unpleasant feeling of being subjected to the gaze of others in a place which is considered intimate and private (4).

Results from generic AAL user studies have shown, however, that barriers related to privacy and intrusion may be disregarded in favor of the benefits leading to the acceptance and adoption of AAL. For instance, the results of Ehrari et al. (28) show that older adults are sometimes willing to override privacy concerns to achieve more safety and autonomy by having monitoring technology installed. This is particularly the case when they feel in control of the situation. Similarly, Schomakers and Ziefle (21) report that privacy-related barriers are traded-off in favor of security-related benefits as long as the system is reliable. Other reasons for a more favorable and accepting attitude toward AAL technology are perceptions of higher needs for care (29) and the motivation to avoid being a burden for relatives who are otherwise charged with the care (3, 30) and/or living longer independently in the own home (4, 31).

Keeping the current living situation and seeing the own four walls as a “final home” is strongly desired among older adults (31). Thereby, the connections older adults have to their home are expressed among others through objects and products (32, 33). Regarding these objects, Leonardi et al. (34) report a strict divide between functional objects (e.g., appliances, communication devices) found in the kitchen as well as in the living room, and symbolic objects (e.g., photographs of beloved ones, fittings) placed in the bedroom. In their study (34), the kitchen was seen as the most dynamic and important place in the home concerning daily routine activities whereas the living room had representational, functional, and aesthetic roles for leisure activities and welcoming guests. The bedroom was considered the least dynamic room in terms of changes of furniture and functions but it was seen as the most comfortable room in the house regarding emotional aspects (34). These emotional aspects lead to the subjective perception that the bedroom would be the safest and most secure place in the living environment even though objectively many age-related accidents may happen during sleep or while being tired and in the darkness. In turn, the kitchen was perceived as the most dangerous place in the home because many chores are executed and technological devices are used. Interestingly, the bathroom was not even perceived as part of the home, at least from an emotional point of view (34). Overall, these individual meanings and social implications of staying independently at home and “age in place” may have a considerable weight in these trade-off processes when considering introducing assistive technology in different parts of the elders' homes (31, 35).

Given this entanglement of factors playing a role in AAL technology adoption, one of the main challenges is to match the functioning of technology—video-based AAL in particular—with the life of actual users living with AAL technology in their homes (36) and their willingness to accept these devices in their homes. Thus, in the development of a well-functioning technology, it is essential to understand daily behaviors, i.e., what is done in the own home on a daily basis and how (potential) users feel about having these specific daily activities monitored by video-based AAL technology. Previous research has shown that acceptance of video-based monitoring declines the more private the room in the own home is perceived (26, 27). As Himmel and Ziefle (27) report, video monitoring was rather rejected overall but the living room, the home office, and the kitchen were rated as the most accepted rooms in the home for video monitoring. The bed- and bath-room were least accepted to be monitored by a camera (26, 27).

Complementary to these findings, Caine et al. (37) show that the comfortableness of being monitored with a visual sensor depends on the performed activity. Sensitive and intimate activities such as hygiene care, showering, or sexual activity were evaluated as particularly uncomfortable to be monitored visually (37). Similarly, Maidhof and Hashemifard et al. (38) report that the comfortableness of being monitored visually decreases the more skin is shown. Thereby, intimate activities such as washing oneself, showering, changing clothes, or toileting were perceived as uncomfortable to be filmed even when care is needed. With the aim of understanding privacy concerns, Choe et al. (39) researched typical activities and habits people do at home that they would not want to be recorded. Their results revealed that the most frequent among the 1,433 activity descriptions mentioned were related to self-appearance, intimacy, cooking and eating, media use, and oral expressions. When looking at the locations where monitoring is critical, the bedroom was considered the most private place in the home and participants related it to sexual activities, sleeping, and (un-)dressing. The authors even mentioned specific paths in the home with a particular private notion, for instance, the walk from the bedroom to the bathroom and then, perhaps undressed, from the bathroom to the laundry to wash the clothes taken off (39).

Concluding, the sensitivity of rooms within the home is of considerable impact. Some rooms like the bathroom and the bedroom certainly have a more private and intimate character (27, 34) and might therefore be per se less accepted for visual monitoring (26, 27, 40). However, to understand the acceptance of video-based AAL including the weighing up of benefits and barriers, these past findings have also highlighted that it is relevant and reasonable to consider the monitored activities themselves, detached from the space. Especially, intimacy and privacy requirements may change depending on the activity and the skin shown no matter where this activity is taking place in the home (37–40).

Understanding the acceptance activity specifically is also timely from a technological perspective. Large activity recognition datasets are made available which comprise visual data of nonscripted daily activities that show people interacting in the home environment (41, 42). In the context of AAL, such datasets enable the development of effective machine-learning techniques for human activity recognition on motion, action, and activity level (10, 43). From a social science perspective, it is reasonable to assess acceptance and privacy perception on activity level which for computer vision is defined as a sequence of actions that can have a duration of several minutes to several hours and include rather complex interactions between humans and objects in the environment (e.g., preparing breakfast, brushing teeth) (43, 44). Besides, these large datasets are used for the development of methods to preserve the privacy of the body (45–48). These privacy preservation methods involve the use of various visualization modes (i.e., image filters) which make people and their body shapes unrecognizable to varying degrees. Being among few in considering activity-specific acceptance, a recent study by Offermann et al. (49) investigated the acceptance and privacy of video-based AAL technology including the perception of different visualization modes in three specific everyday situations (i.e., eating, undressing, falling). First of all, the results (49) confirmed the general trend of reduced acceptance of video-based AAL technology. Second, the study demonstrated that the three specific situations were perceived differently from each other in terms of privacy and intimacy and, consequently, the preferred data visualization mode varied among the situations as well. The eating scenario was perceived as moderately private, the undressing scenario as highly private and the falling scenario as not private at all. As a consequence, the falling scenario was accepted to be visualized without any privacy-preservation methods for most of the participants whereas most participants rejected video-based monitoring during the undressing scenario. Overall, these findings show that acceptance is highest and privacy concerns lowest in a safety-critical moment such as a fall. However, these accidents are not foreseeable and are very likely to happen, for instance, exactly during intimate activities (e.g., slipping in the shower, falling while dressing, respiratory, or coronary problems during sleep) or even during basic household chores (e.g., cutting the finger while cooking, falling while cleaning windows).

Therefore, to contribute to the still very little researched field of video-based AAL technology acceptance this study focuses on investigating, first the privacy need for a variety of daily living activities (ADLs) and second the evaluation of acceptance parameters for the visual monitoring of three distinct activities of daily living during which a safety-critical accident happens. In line with previous investigations, these three assessed ADLs vary in their degree of privacy and intimacy.

## 2. Materials and methods

The empirical approach of this study is described in this chapter, including the research aim, the explanation of the design of the online survey, and its subsequent data analysis. In addition, the sample is presented.

### 2.1. Objective and aim of the study

As described before, knowledge about the acceptance and perception of using video-based AAL technologies in daily life is still sparse. In particular, it is so far not well elaborated if different activities of daily living need distinct levels of privacy preservation. Meeting the demands of everyday life including different activities of daily living, it should be investigated whether the acceptance and perception of using video-based AAL technology in daily life are influenced by specific types of activity of daily living. In this regard, activity types should be compared that differ in their degrees of intimacy and privacy needs accordingly.

Based on that, the underlying research questions were the following:

1. How much privacy is required for a variety of activities of daily living? (**RQ1**)
2. Does the acceptance i.e., behavioral intention to use video-based AAL technology, including evaluations of benefits and barriers, significantly vary depending on the activity of daily living monitored (the distinction between Household, Social and Intimate Activity)? (**RQ2**)

### 2.2. Questionnaire design

The questionnaire was developed based on both, the previously mentioned literature on acceptance and privacy perceptions of AAL technology (3, 14, 20, 25, 50) and a preceding qualitative study. This qualitative study consisted of 10 semi-structured interviews in Germany (5 males, 5 females, age range: 24–61). During the first part of these interviews, typical activities of daily living were gathered. Therefore, participants were given a ground plan of an apartment and had to name all the activities that came to their minds and rank all mentioned activities according to how much privacy they would need. In the second part of the interviews, video-based AAL technology was introduced and participants were asked about their opinions (e.g., advantages, disadvantages, and conditions for usage) on these devices in their homes in older age during different scenarios (i.e., household activity, social interaction, intimate activity). The interviews were audio-taped, transcribed verbatim, and then analyzed using the theoretical framework of content analysis described by Mayring and Fenzl (51). The obtained qualitative results formed the basis for the items used in the quantitative questionnaire. More specifically, the list of activities of daily living as well as items for benefits and barriers were generated from the qualitative study. After the development of the questionnaire, it was delivered exclusively online addressing a random sample. All constructs and items including their metrics are presented in Appendix 1.

**Figure 1:**
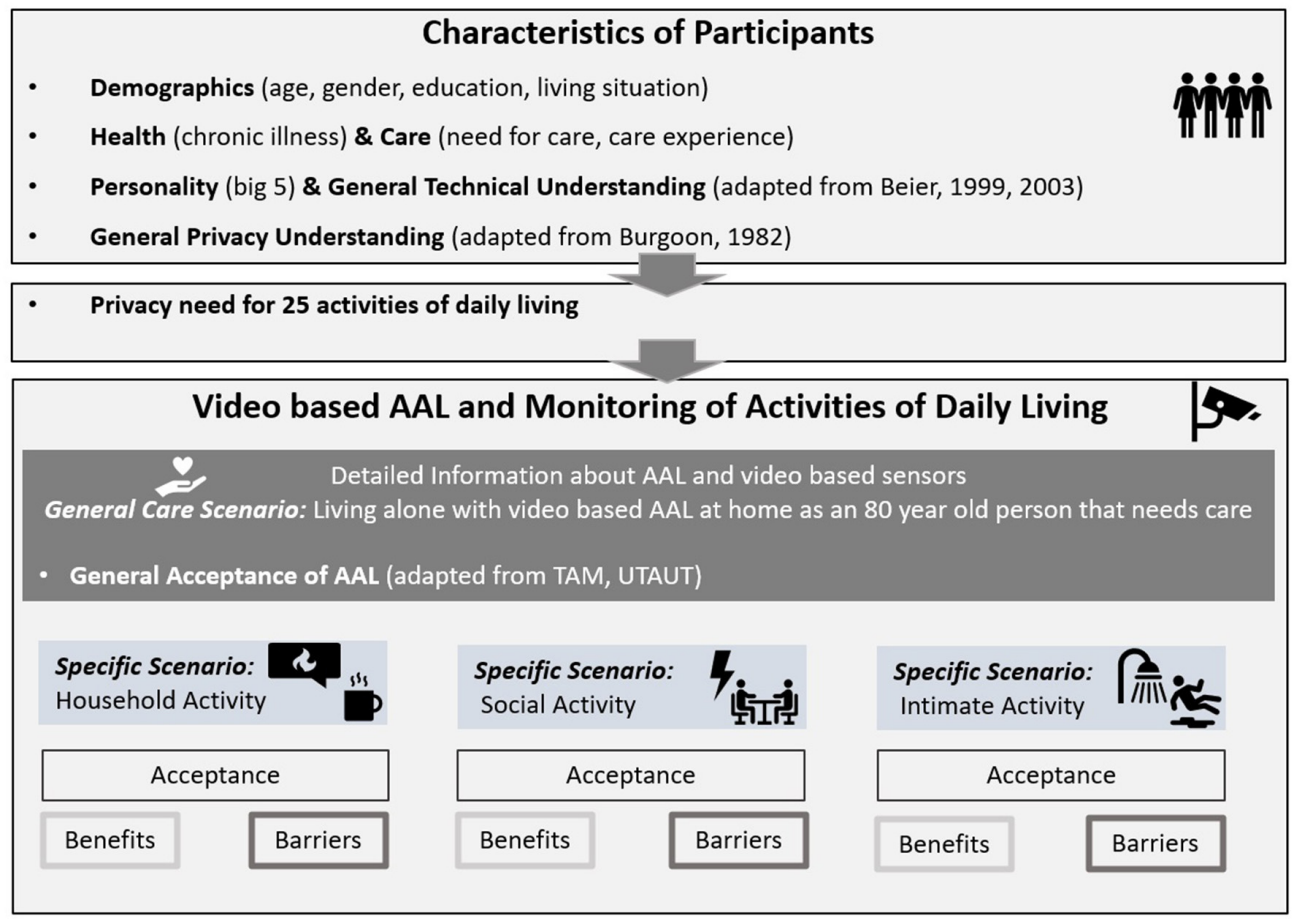
Empirical design.

The questionnaire consisted of two main parts and is illustrated in Figure 1. The first part of the questionnaire started with demographics, such as age, gender, educational level, as well as the living situation and place of living. Then, information about participants' health (i.e., if they suffered from a chronic illness and needed care) and about having experience in caring for another person was asked. Subsequently, additional user factors such as working field, and technical understanding (four items) (52, 53), and psychometrics were assessed. In addition, general privacy attitudes [16 items, partly based on (54)] were assessed to evaluate participants' understanding of the meaning of privacy in daily life. Then, a list of 25 typical activities of daily living was presented and participants had to indicate on a four-point Likert scale (1 = very little privacy, 4 = very much privacy) how much privacy these different activities of daily living require. The second part of the questionnaire introduced video-based AAL with a detailed explanation. Participants were then asked to evaluate their overall acceptance (eight items) [partly based on (17, 50, 55)] of imagining themselves living with such technology. Three specific activities of daily living happening in the own home were presented (described in more detail below) and evaluations regarding the specific acceptance of monitoring the activity [operationalized as the behavioral intention (three items)], perceived benefits (five items) and barriers (six items), as well as technological preferences, were assessed for each scenario. At the end of the questionnaire, participants could write their feedback and/or critiques regarding the questionnaire in an open field. If not described otherwise above, all scales were rated on six-point Likert scales (1 = completely disagree, 6 = completely agree).

To understand participants' attitudes toward monitoring different activities typically performed during the day, three scenarios were introduced in which video-based AAL technology actively supports the person in need of care because a safety-critical accident happens. The scenarios were presented in randomized order to control for sequence effects.

One scenario targeted basic chores such as cooking, cleaning, or tidying up during which a fire is caused and detected by the monitoring camera. This scenario is labeled as *Household Activity*. Another scenario focused on longer interactions with other persons such as chatting or playing with friends or grandchildren. Due to these interactions, medicines are forgotten and cause a deviation from normal healthy behavior which is then detected by the video system. This activity is named *Social Activity*. Lastly, one scenario refers to activities typically requiring a certain amount of physical stability and strength as well as the showing of skin, such as showering, toileting, or changing clothes. Because of physical decline, a fall is happening and detected by the monitoring system. This scenario is labeled as *Intimate Activity*.

### 2.3. Participants

The targeted number of participants was 150 which was reasonable given the resource constraints (56). Overall, *N* = 202 people opened the questionnaire. It was delivered exclusively online through the social networks of the research team following a convenience sampling approach. As part of the data cleaning process, data from 51 participants who just opened the questionnaire were deleted immediately and data from another four who completed < 75% of the questionnaire were excluded from the analysis. Eventually, data from *N* = 146 participants was used for data analysis which almost met the targeted sample size.

The majority of participants were recruited in Germany (*n* = 102) and a smaller part was recruited in Bulgaria (*n* = 44). Participants' age range was from 17 to 81 (M = 37.02; SD = 16.32) with slightly more females (66.7%; *n* = 94) than males (33.3%; *n* = 47; no participants indicated being diverse or disclosed information). Asked for participants' highest educational degree, 6.4% (*n* = 9) participant had at least a secondary school diploma or 27.7% (*n* = 39) a high school diploma/A-Level degree. The remaining 65.9% either had a university degree (*n* = 89; 63.1%) or a promotion/doctoral degree (*n* = 4; 2.8%). A slightly smaller fraction, namely 35.5% (*n* = 50) indicated having a technical profession compared to 64.5% (*n* = 91) not working in a technical environment. Overall the sample showed a quite decent understanding of technology (M = 4.13; SD = 0.91; Cronbac's α = 0.73). The majority (66.0%; *n* = 93) said to live in the city, only 16.3% (*n* = 23) in the suburbs and slightly more, 17.7% (*n* = 25), indicated living in the countryside. Some participants (18.4%; *n* = 26) lived alone, 43.3% (*n* = 61) shared their living space with another person such as a partner and the remaining 38.3% (*n* = 54) stated to live together with more than one person such as a family or flatmates. The sample was quite healthy with only about a quarter of participants, namely 24.1% (*n* = 34) suffering from any chronic illness (i.e., asthma, migraine, endometriosis, high blood pressure, or diabetes). A small fraction needs care in their daily life (6.4%; *n* = 9). Some participants indicated having experience in caring for another person, either professionally (12.8%; *n* = 18) or informally (34.8%; *n* = 49; professional and informal care experience can overlap).

### 2.4. Statistical analysis

For statistical analysis regarding the influence of the activity type on technology acceptance and on technological preferences, repeated measures analyses of variance (rmANOVA) were applied, and the significance value in the multivariate tests was taken from Wilks' Lambda. Furthermore, a possible interaction between activity type and acceptance evaluations was analyzed. If the assumption of sphericity was violated (Mauchly's Test of Sphericity < 0.05), Greenhouse-Geisser correction or Huynh-Feldt (HF) was utilized. The statistical significance level was always set at the conventional level of 5%. Non-significant results are labeled as n.s. (not significant). For effect sizes, eta squared (η^2^) was calculated. For descriptive analyses, means (M), and standard deviations (SD) are reported.

## 3. Results

The results are presented in line with the research questions, starting with the evaluations of privacy needs for several activities of daily living. Then, results on privacy parameters regarding video-based AAL monitoring are reported.

### 3.1. Privacy need in daily life (RQ1)

The first research question refers to privacy in daily life, independent of any monitoring technology. Therefore, participants had to evaluate to what extent they agreed that several definitions of privacy (i.e., about the physical environment, about their own identity and personal thoughts as well as regarding confidential data and about social interactions) actually describe the concept of privacy for them. Overall, privacy understanding was elaborate (16 items; M = 4.80; SD = 0.60; Cronbach's α = 0.85) as participants overall rather agreed to the single definitions.

**Figure 2:**
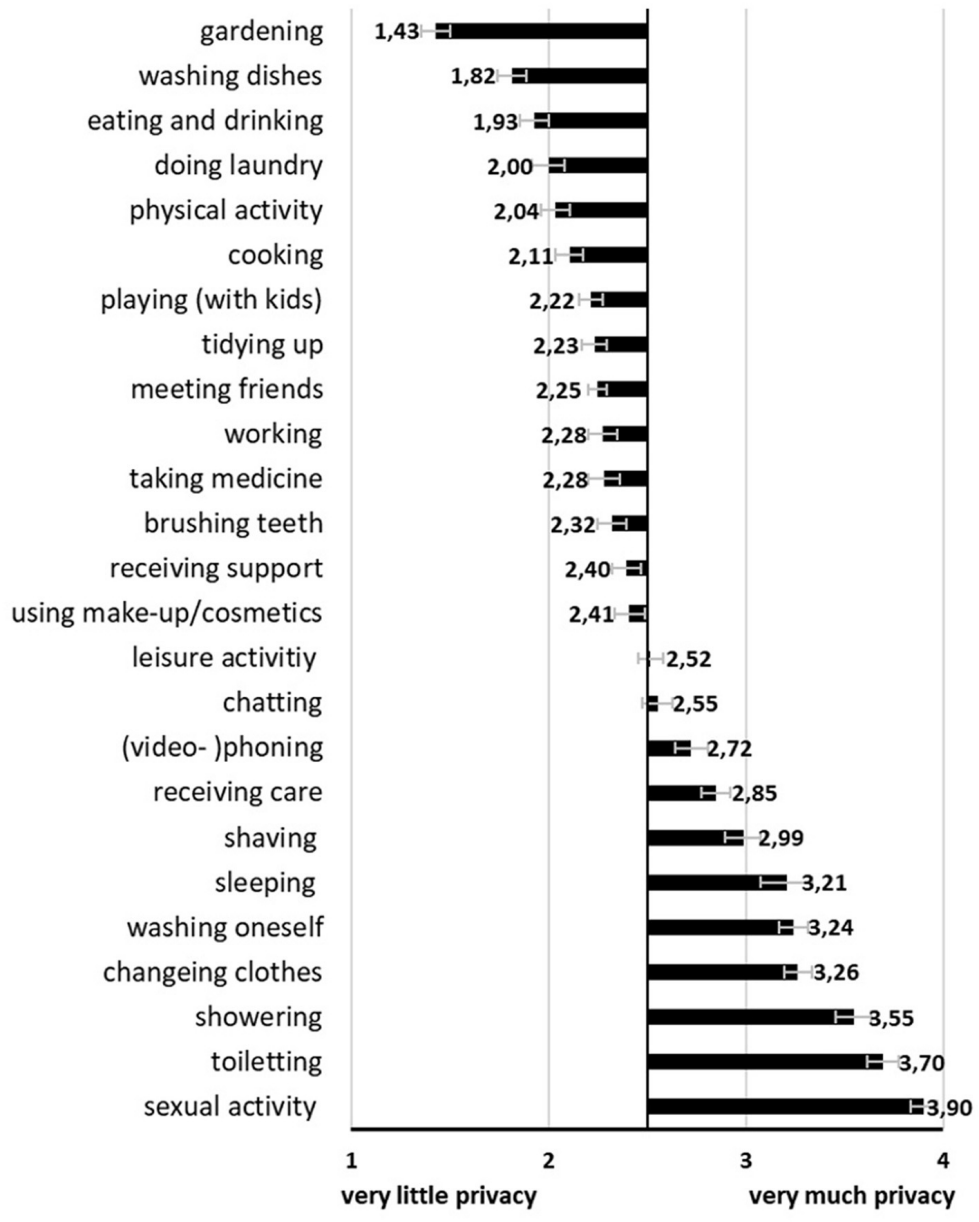
Evaluations of privacy need for 25 activities of daily living (Mean values adjunct to bars. Error bars show standard error).

In the second step, we gathered an overview of how much privacy is needed for each of the listed 25 activities of daily living (1 = very little privacy; 4 = very much privacy). These activities were collected during a previous qualitative assessment and are supposed to cover a broad range of activities (older) adults normally perform during the day. Overall, the common trend shows that typical household routines like gardening (M = 1.43; SD = 0.68) or washing dishes (M = 1.82; SD = 0.88) require the least privacy while intimate activities like showering (M = 3.55; SD = 0.67), toileting (M = 3.70; SD = 0.58), or sexual activity (M = 3.90; SD = 0.30) need the most privacy. Social activities such as meeting friends (M = 2.25; SD = 0.90), leisure activity (M = 2.52; SD = 0.87), or chatting (M = 2.55; SD = 0.83) range in between the extremes of very little privacy and very much privacy. Similarly, care activities like taking medicine (M = 2.28; SD = 0.99), receiving support (M = 2.40; SD = 0.86), and receiving care (M = 2.85; SD = 1.07) are evaluated as requiring medium privacy. These results are visualized graphically in Figure 2.

### 3.2. Acceptance parameters of video-based AAL during different activities of daily living (RQ2)

As part of the second research question, video-based AAL was introduced, the general acceptance was assessed and then reference was made to three different scenarios of daily living. For each scenario, we looked at participants' specific acceptance, i.e., the behavioral intention to use such camera systems and their rating of benefits and barriers during a particular activity. Statistical details can be viewed in Table 1.

**Table 1:** Statistical results for effects of activities of daily living on acceptance (F-statistics refer to tests of within-subjects effects).

	**Household activity**	**Social activity**	**Intimate activity**	**Statistic of differences**	**Level of significance**	**Effect size**
**Construct**	**M (SD)**	**M (SD)**	**M (SD)**	***F*** **(within subjects)**	***p***	η^2^
**Behavioral intention**	**4.07 (0.62)**	**3.78 (0.74)**	**3.57 (0.79)**	***F***_**(1.9, 284)**_ **= 25.794**	**0.000**	**0.154**
**Overall benefits**	**4.64 (0.86)**	**4.51 (0.83)**	**4.41 (0.91)**	***F***_**(2, 282)**_ **= 8.437**	**0.000**	**0.056**
Gain in safety	4.99 (0.99)	4.23 (1.22)	4.63 (1.21)	*F*_(2, 276)_ = 11.765	0.000	0.079
Increased independence and autonomy	4.64 (0.86)	4.51 (0.83)	4.41 (0.91)	*F*_(2, 280)_ = 0.928	0.396 (n.s.)	0.007
Faster reactions in emergencies	4.64 (0.86)	4.51 (0.83)	4.41 (0.91)	*F*_(1.9, 258.3)_ = 4.39	0.016	0.030
Gain in comfort and convenience	4.64 (0.86)	4.51 (0.83)	4.41 (0.91)	*F*_(2, 276)_ = 5.798	0.003	0.040
Relief for caring relatives	4.64 (0.86)	4.51 (0.83)	4.41 (0.91)	*F*_(2, 274)_ = 0.959	0.385 (n.s.)	0.007
**Overall barriers**	**3.82 (0.99)**	**4.05 (0.92)**	**4.13 (0.90)**	***F***_**(1.9, 270.7)**_ **= 16.620**	**0.000**	**0.105**
Invasion of privacy	4.64 (0.86)	4.51 (0.83)	4.41 (0.91)	*F*_(1.7, 243.6)_ = 1.904	0.000	0.101
Fear of data misuse	4.64 (0.86)	4.51 (0.83)	4.41 (0.91)	*F*_(1.9, 263.7)_ = 14.314	0.000	0.093
Sense of surveillance	4.64 (0.86)	4.51 (0.83)	4.41 (0.91)	*F*_(1.9, 260.4)_ = 11.492	0.000	0.076
Fear of technical problems	4.64 (0.86)	4.51 (0.83)	4.41 (0.91)	*F*_(1.9, 265.2)_ = 0.658	0.513 (n.s.)	0.005
Fear of false alarms	4.64 (0.86)	4.51 (0.83)	4.41 (0.91)	*F*_(1.9, 271.5)_ = 0.058	0.941 (n.s.)	0.000
Feeling of incapacitation	4.64 (0.86)	4.51 (0.83)	4.41 (0.91)	*F*_(2, 278)_ = 6.888	0.001	0.047

n.s. means non significant.

Independent from the activity and in general, video-based AAL technology in the own home was slightly accepted overall (M = 3.79; SD = 0.93; Cronbach's α = 0.86) based on the mean of the scale (M = 3.5).

**Figure 3:**
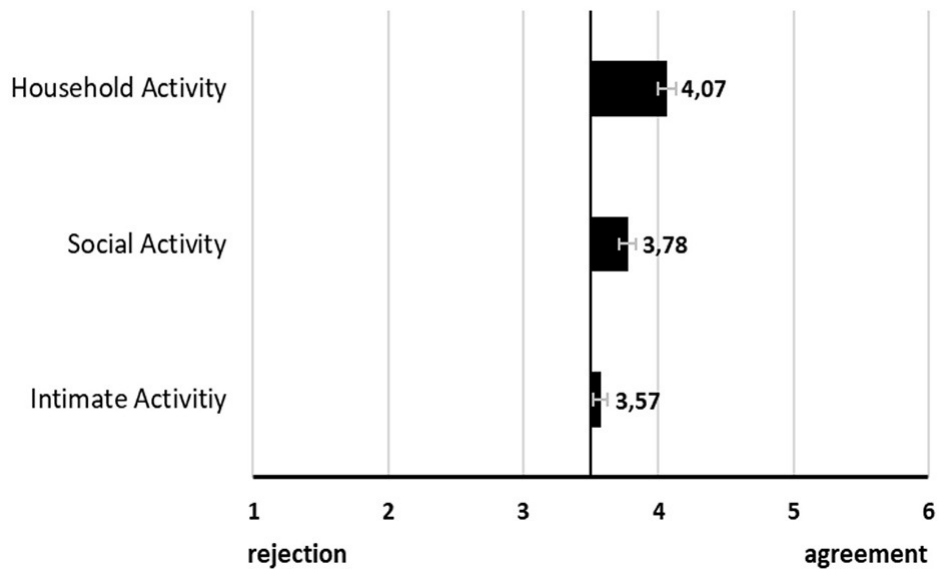
Visualization of specific acceptance of video-based AAL monitoring (Mean values adjunct to bars. Error bars show standard error).

Regarding activity-specific evaluations, there was a significant effect of activity type on the specific acceptance of using video-based AAL technology in terms of the behavioral intention of use (see Figure 3). The behavioral intention to use video-based AAL was highest for household activity and lowest for intimate activity. When looking at the details, *post-hoc* comparisons revealed significant differences between two of the three activity types, namely between household (M = 4.07, SD = 0.62) and social activity (M = 3.78, SD = 0.74, *p* < 0.000) as well as between household and intimate activity (M = 3.57, SD = 0.79, *p* < 0.000).

**Figure 4:**
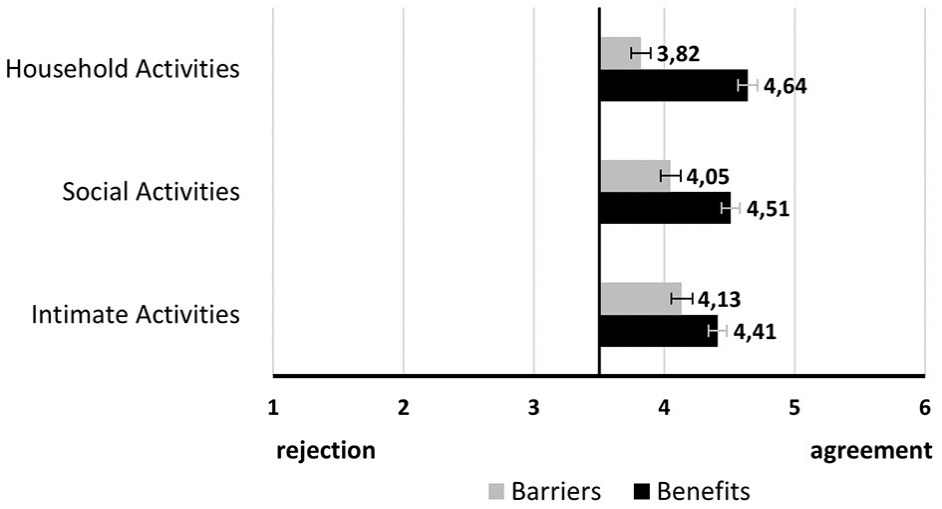
Visualization of benefits and barriers overall (Mean values adjunct to bars. Error bars show standard error).

The overall benefits and overall barriers are the mean scores of all single mean scores of benefits and barriers, respectively. Similar to the behavioral intention, for both overall benefits and barriers scores, there was a significant effect of activity type and again, benefits for the household activity were rated highest and benefits for the intimate activity lowest. The inverse was the case for overall barrier scores (see Figure 4). *Post-hoc* comparisons of perceived benefits showed a significant difference only between household activity (M = 4.64, SD = 0.86) and intimate activity (M = 4.41, SD = 0.91 *p* < 0.000) whereas *Post-hoc* comparisons of barriers revealed a significant difference between household (M = 3.82, SD = 0.99) and social activity (M = 4.05, SD = 0.92, *p* < 0.000) as well as between household activity and intimate activity (M = 4.13, SD = 0.90, *p* < 0.000).

**Figure 5:**
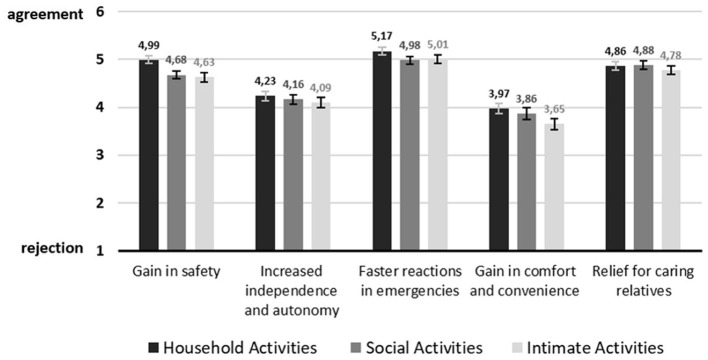
Visualization of benefits on item level (Mean values adjunct to bars. Error bars show standard error).

Zooming into single benefits illustrated in Figure 5, for three of the five benefits, namely, gain in safety, faster reactions in emergencies and gain in comfort and convenience, there was a significant effect of activity type. Interestingly, household activity was always rated differently from either both or at least one of the two other activities. Social activity and intimate activity did not significantly differ from each other in their evaluations. *Post-hoc* comparisons for gain in safety revealed that household activity (M = 4.99, SD = 0.99) was evaluated significantly differently from social activity (M = 4.68, SD = 1.02, *p* < 0.001) and intimate activity (M = 4.63, SD = 1.21, *p* < 0.000). While for increased independence and autonomy, no significant differences were found, there was a significant difference in faster reactions in emergencies and especially between household activity (M = 5.17, SD = 0.91) and social activity (M = 4.98, SD = 0.91, *p* < 0.016) and between household activity and intimate activity (M = 5.01, SD = 0.98, *p* < 0.033). A difference in evaluation for gain in comfort was only significant between household activity (M = 3.97, SD = 1.30) and intimate activity (M = 3.65, SD = 1.37, *p* < 0.003). Relief for caring for relatives was not significantly evaluated differently.

**Figure 6:**
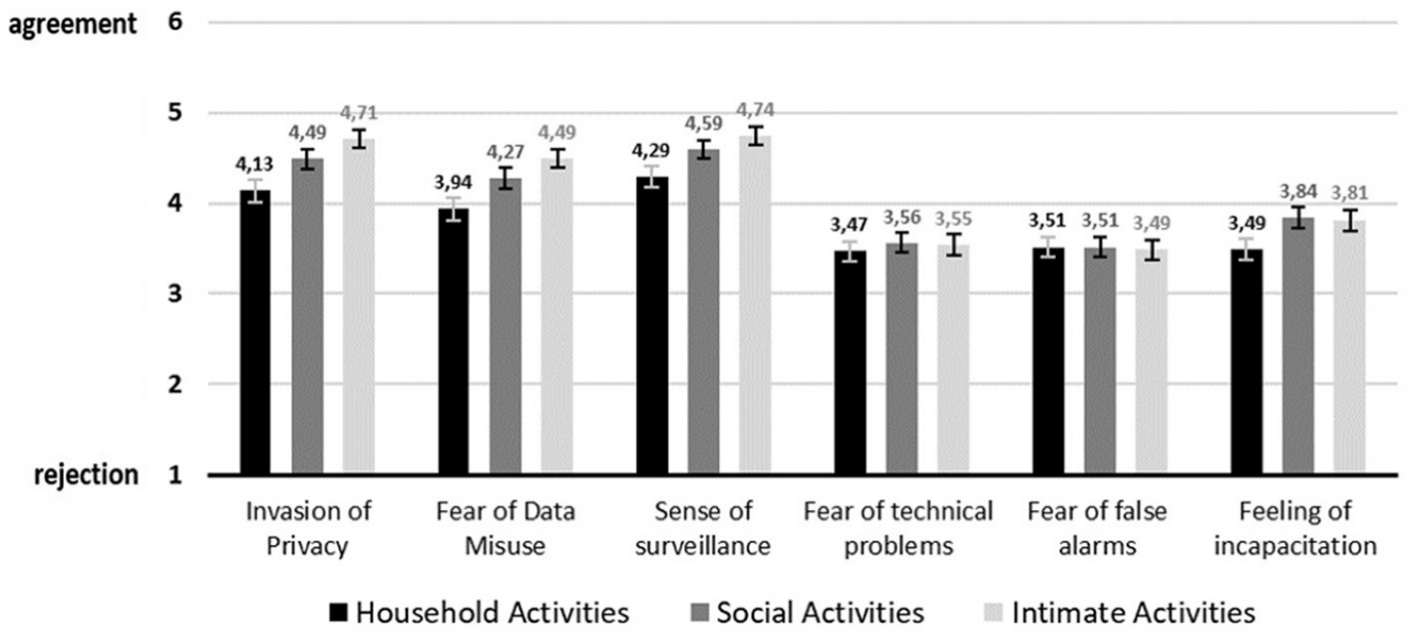
Visualization of barriers on item level (Mean values adjunct to bars. Error bars show standard error).

Among the single barriers visualized in Figure 6, invasion of privacy, fear of data misuse, sense of surveillance, and feeling of incapacitation revealed a significant effect on activity type. One more time, household activity was always rated differently than either social and intimate activity or at least one of them. Unlike the benefits, the barriers in social activity and in intimate activity did differ significantly regarding two evaluations of privacy concerns. More specifically, for invasion of privacy depicted a significant difference between all three activities, namely between household activity (M = 4.13, SD = 1.44) and social activity (M = 4.49, SD = 1.27, *p* < 0.005) and between household activity and intimate activity (M = 4.71, SD = 1.25, *p* < 0.000) as well as between social activity and intimate activity (*p* < 0.016). Similarly, fear of data misuse was rated significantly different between all three activities. As such, between household activity (M = 3.94, SD = 1.51) and social activity (M = 4.27, SD = 1.38, *p* < 0.009) and between household activity and intimate activity (M = 4.49, SD = 1.27, *p* < 0.000) and again between social activity and intimate activity (*p* < 0.046). *Post-hoc* comparisons for the sense of surveillance showed a significant difference between evaluations of household activity (M = 4.29, SD = 1.40) and social activity (M = 4.59, SD = 1.27, *p* < 0.006) as well as between the evaluations of household activity and intimate activity (M = 4.74, SD = 1.22, *p* < 0.000). While the two barriers concerning technical issues were different, the feeling of incapacitation was evaluated significantly differently for household activity (M = 3.49, SD = 1.35), and social activity (M = 3.84, SD = 1.34, *p* < 0.002) and between household activity and intimate activity (M = 3.81, SD = 1.42, *p* < 0.013).

## 4. Discussion

In this section, key insights of the study are discussed and their relation to the existing research is outlined. If applicable, several recommendations for technology development are proposed. Lastly, the limitations of the study are described and future research ideas are proposed.

### 4.1. Key insights and implications

As part of the first research question (RQ1), the amount of privacy needed for the performance of various activities of daily living was investigated. In line with previous research (5, 37, 38), the need for privacy differs between single activities. A general trend can be observed starting with a low privacy need for basic household chores, a medium privacy need for social activities and care-related ones and the highest privacy need for the most intimate activities. The existence of two extreme poles, uncritically perceived household activities compared to highly critically perceived intimate activities has been reported before (37, 38, 40, 49) in the context of video-based AAL technology and is confirmed within the results of the current study. Except for cooking and eating which were counted among the most privacy concerning activities in the study of Choe et al. (39), the current findings are very much comparable. These different evaluations for cooking and eating may be due to measurement differences across studies. Other research assessing the privacy of rooms reported the kitchen as the least private room and the most accepted room for (visual) monitoring (26, 27, 34) which again matches the current results. Our findings show that kitchen-related activities such as washing dishes, eating and drinking, and cooking are among the activities requiring the lowest privacy need. However, more privacy-critical activities are also very likely to happen in the kitchen such as chatting, (video)-phoning or receiving care. This nuanced overview further confirms that it was reasonable to assess privacy needs on the activity level rather than to assess privacy needs for single rooms only—especially, when considering monitoring these activities. In fact, if just the privacy of the room were assessed, we would not have empirically understood that brushing teeth needs much less privacy compared to other typical bathroom activities.

In addition, these findings are relevant for computer vision interested in developing methods to protect privacy during video-based assistive monitoring (45, 46, 57, 58). Prominent datasets like the EPIC-kitchen (41) show typical kitchen activities and allow the development of methods for recognizing single actions and activities through computer vision. According to the current findings, usual kitchen activities before eating (i.e., cooking) are considered more private than activities after eating (i.e., eating/drinking and washing dishes). During the more privacy-critical meal preparation phase the use of wrapped food with visible brand names, the quality and quantity of (potentially unhealthy) ingredients, and the continuous opening of cupboards are privacy-related aspects which are much less relevant at a later stage. These aspects do not relate to the person or body itself but to the objects used during these cooking activities. Therefore, in such a setting, one effective method to preserve privacy in the visual output may be to provide filters for (branded) objects in the human's hand and in the cupboard, especially when meal-preparing actions are performed (likely to happen directly after entering the kitchen). Hasan et al. (59) proposed a cartooning method to replace personal objects and items with clip-art images and reported positive user feedback in a subsequent study (60). For privacy preservation, during the monitoring of kitchen activities, this approach may be reasonable as well. When looking at the more privacy-critical activities it becomes clear that intimate activities involve some level of nudity (physical and psychological nudity). Thereby, psychological nudity mainly regards activities where the self is on display like receiving care or talking on the phone or during a chat. Physical nudity refers to naked body parts shown during the mentioned bathroom activities like showering, or toileting, for example. The showing of skin is perceived as highly uncomfortable and critical to be monitored visually (37, 38) and the current findings confirm the high privacy need for these activities. Thus, nudity detection methods should be further improved and targeted to activities such as showering or toileting. The identification of a specific action or activity through computer vision may comprise the detection of objects used in the activity. Across intimate activities assessed in this study, the only obvious common denominator is, next to the bathroom location, the involvement of water (i.e., liquid) and water faucets. This may be a hint for improving activity recognition methods, even though it is a rather weak proposal.

The second research question (RQ2) specifically investigated the acceptance parameters of video-based AAL for three distinct activity scenarios in which an unforeseen and safety-critical accident happened. Results show that the behavioral intention to use video-based AAL technology was never completely rejected but it was not even strongly accepted either. The fact that acceptance for video-based AAL technology was slightly more positive than assessments in earlier studies (13, 26, 27) could be referred to the safety-criticality of each situation. In the case of safety-critical situations in which urgent help is vital, e.g., in an accident scenario (i.e., a fall), privacy needs diminish (49). In this study, we presented three different scenarios with comparable safety-criticality. Even though the technology was evaluated positively in terms of acceptance - even during an intimate activity—the behavioral intention to use them changed significantly depending on the situation. The household activity received the highest acceptance rates whereas the intimate activity had the lowest. Thus, we can conclude that any safety-critical incident changes the perception of video-based AAL toward a more positive evaluation—even during intimate activities. However, the graduations of uncomfortableness and criticality of filming intimate activities do persist and are particularly pronounced during intimate activities—despite the safety aspects (37, 38). One cautionary note needs to be added here: The conclusion that whenever there is a real danger to life, everyone would tend to suspend all privacy and protection requirements and agrees to video-based AAL technology falls short. Whether and when users and older users in particular allow AAL technology even in critical situations depends on their attitudes to life and end-of-life decisions (61, 62). Of course, it is also reasonable to assume that cameras are rejected simply because people do want to have control in life-end decisions, not wanting that these decisions are prevented by camera sensors that call automatically for help not asking if the person wants help (63). Here, future research must also include the question of who may have control over living and dying—and this is certainly a question related not only to person-specific factors but also to cultural and social values (64, 65).

As part of the acceptance research on AAL technologies, the benefits and barriers to adopting these assistive devices are usually identified and assessed (3, 14, 25). In this study, overall and across all three activities the benefits of using video-based AAL received higher agreement than the barriers. This pattern may have, in part, lead to the overall accepting trend regarding the behavioral intention of using these visual devices (3, 12, 14, 21).

Still, the benefits and barriers were rated differently across the single activity scenarios. Monitoring during household activity received the highest agreement in terms of benefits and the lowest agreement in terms of barriers. The opposite was the case for intimate activity. Interestingly, this trend is similar to the previously described evaluation of privacy needs for several activities of daily living, in which household and intimate activities represent two poles. On one hand, visual monitoring of household activities seems to be welcomed and is perceived as rather unproblematic regarding typical barriers such as privacy concerns or technical issues. However, in the case of intimate activities, these barriers almost balance out the benefits making it the most critical activity to be monitored visually. These findings can be connected to previous investigations regarding older adults' domestic spaces (34). Many household activities happen in the kitchen which is therefore the most functional and most dangerously perceived place in the home. Consequently, assistive technology, including camera sensors, is most accepted there, as several studies have shown (26, 27, 34). Intimate activities tend to happen in the bedroom or in the bathroom and are highly critical to be monitored due to privacy concerns (11, 27, 38). These privacy concerns may have led to low acceptance rates regarding typical activities in the bathroom, the most unimportant and unemotional place in the house (34). In turn, the bedroom is considered the most intimate and secure place. Hence, besides privacy concerns, the subjective need for being monitored in this already safe place may have been perceived as low (34). Overall, given this pattern, we may use an analogy of a continuum to describe the acceptance, comfortableness and privacy of monitoring activities with camera sensors. Thereby, household activities reside on the positive, most accepted and least privacy-critical end of the continuum and intimate activities on the opposite, negatively, less accepted perceived ending. This duality in the acceptance parameters is further highlighted when looking at the benefits and barriers on the item level. Most of the time, the benefit and barrier evaluation of the household activity differed significantly from the evaluation of the intimate activity. Evaluations of the social activity however did not always show significant differences. More in detail, social activity was frequently evaluated similarly to intimate activity. Hence, within this analogy of a continuum, monitoring of social activity oscillates in the middle but tends mostly toward the more critical and less accepted end-pole.

Regarding the single benefits, *faster reactions in emergencies* was the most relevant benefit across all three activities. This is in line with a recent study dealing with video-based AAL of Offermann et al. (49) that also reported emergency-related aspects as the most important benefit. Related to this, another security-related benefit, namely *gain in safety* was the second most relevant benefit for household activity and the third most important for social and intimate activity. This fits with the overall trend in AAL user research reporting the safety and security aspect as one of the main benefits of using AAL technology (3, 12, 14, 21). Evaluations for *increased independence and autonomy* and *relief for caring relatives* were not rated differently across the activity situation. In a state of physical decline, both benefits may be equally valid for all parts of the home (20, 31).

When looking at the barriers an interesting and almost identical pattern emerges for the privacy-related barriers of *invasion of privacy, fear of data misuse* and *sense of surveillance* across the three activities. Privacy issues are lowest for household activity and highest for intimate activity, thus, become increasingly relevant the more intimate the situation is. Again, this is in line with the previously described continuum and with previous findings reporting privacy issues as the most important barrier for visual monitoring (4, 6, 10, 11, 13). Compared to these privacy-related barriers, the barriers regarding technical issues become much less relevant.

### 4.2. Limitations and future work

This study provided novel insights into the impact of specific activities of daily living on the perception and acceptance of video-based AAL technology. Beyond that, the methodological approach, content, and sample of this study have limitations which should be considered for future research in this field.

Starting with the sample in this study, rather young and highly educated participants were reached. While these are a relevant target group of future potential users, they consist of only one among several possible target groups. As video-based AAL technologies have a promising potential to support even older and frail people, future studies should try to focus more on this specific group of future users. Further, the participants originated from Bulgaria and Germany. This would enable a country-based comparison which was not focused on in this investigation. As privacy regulations and perceptions differ strongly across different countries and cultures, future research should try to realize broader samples comparing participants from different countries and origins.

Considering methodological aspects, the first limitation refers to the scenario-based approach. The applied scenarios representing the different activity types provided the basis for experimentally varying the evaluations of acceptance and the perception of relevant benefits and barriers. However, we cannot exclude that the evaluations might lead to different agreements or rejections in real-life contexts according to the well-known gap between attitudes and behavior (66). Therefore, future research should realize experiments and user studies focusing on hands-on experience with actual technology or at least specific data output. A second aspect refers to the length of the online survey. Due to the three different scenarios, the design and answering of the survey may have been repetitive. Applying a randomized design, the probability of recurrence effects has been minimized. However, dropout rates and feedback from the participants showed that answering the same items for three scenarios did not represent a desired and optimal format, but allowed the purpose of a direct comparison. For future research, it is not recommended to compare more than two different scenarios based on several constructs and their respective items.

Taking the investigated research questions into account, the results suggest different interesting anchor points for future work. First, we assessed the different needs for privacy based on a selection of typical activities performed during the day. Taking cooking as an example (which resulted in low privacy needs), it represents a combination of many small actions (e.g., cutting food, washing food, heating food up, searching for ingredients in the cupboard). In addition to that, we assessed washing dishes, which resulted in even lower privacy needs, but could have been considered as part of cooking. It would therefore be interesting to understand if the evaluation of privacy needs decreases the more detailed the activity is described and if yes if it is an overall phenomenon (e.g., applicable for intimate activities as well). As a second anchor point, it would be interesting for computer vision to investigate and understand which specific objects are involved in intimate situations being perceived as private. Here, it would be of utmost importance to analyze privacy perception and in particular different facets of data privacy in more detail. This would be informative for supporting the development of activity recognition methods targeting intimate activities and the respective needs of future users in specific. As a last aspect for future research, our study in line with previous research (49) revealed that monitoring of accidents seems to be accepted overall, but to varying degrees depending on what activity is being performed. Given the rather rejecting trend of visual monitoring future work should corroborate this finding. Therefore, it is recommended to deepen the research on acceptance and perceptions of video-based AAL technology in this regard by considering an assessment of accidents with different gravity levels and their interplay with various activities. Based on such insights, concrete recommendations can be derived to inform and support the user-centered development of video-based AAL technology.

## Data availability statement

The raw data supporting the conclusions of this article will be made available by the authors on reasonable request.

## Ethics statement

The studies involving human participants were reviewed and approved by Ethics Committee (Division 7.3) Empirical Human Sciences at the Faculty of Humanities at RWTH Aachen University (Ethical approval number: 2022_12_FB7_RWTH Aachen). The patients/participants provided their written informed consent to participate in this study.

## Author contributions

CM and JO: conceptualization, methodology, validation, formal analysis, investigation, and data curation. CM, JO, and MZ: resources and writing—review and editing. CM: writing—original draft preparation and visualization. MZ: project administration and funding acquisition. All authors have read and agreed to the published version of the manuscript.
