# Supplementary material for: Eyes on privacy: acceptance of video-based AAL impacted by activities being filmed
Source: Front Public Health. 2023 Jul 4;11:1186944. doi: 10.3389/fpubh.2023.1186944 (PMC10352951; doi:10.3389/fpubh.2023.1186944)
Supplement: Supplementary file 1 [file Data_Sheet_1.PDF]

Appendix 1: Overview of all applied constructs, items, and their metrics.

Construct	Item	Origin	Reliability
Technical Understanding (4 Items)	I am interested in understanding the reasons for my actions.	adapted from Beier, 1999	α =.73
	I believe certain things so that others will like me.	adapted from Beier, 1999	
	I like to figure out my feelings.	adapted from Beier, 1999	
	I often put myself under pressure.	adapted from Beier, 1999	
Privacy Attitudes (16 Items)	Controlling the physical proximity of others to myself.	adapted from Burgoon, 1982	α =.85
	A protected zone that keeps others from seeing, hearing, or knowing what is going on inside of me.	adapted from Burgoon, 1982	
	The moment in the bathroom when I take a shower.	based on qualitative study	
	Situations where I withdraw and just be alone.	based on qualitative study	
	Control over my personal information.	adapted from Burgoon, 1982	
	The right to determine how, when, and to what extent data about myself is shared with others.	adapted from Burgoon, 1982	
	If someone entrusts me with something, I keep it to myself.	based on qualitative study	
	My data on my cell phone.	based on qualitative study	
	The ability to think freely and develop an individual identity.	adapted from Burgoon, 1982	
	To determine with whom and under what circumstances to share thoughts and feelings and seek advice.	adapted from Burgoon, 1982	
	In daily life, I can do anything and am free to think and do what I want.	based on qualitative study	
	Others cannot determine for me.	based on qualitative study	
	The ability to withdraw from social interaction.	adapted from Burgoon, 1982	
	The control I have over my social contacts.	adapted from Burgoon, 1982	
	The relationships I have with my family.	based on qualitative study	
	To decide with whom I start a conversation.	based on qualitative study	
Acceptance of Video-Based AAL Technology (3 Items)	I can well imagine the use of video-based technology here.	adapted from Davis, 1989	Overall: α =.86 Household Activities: α =.75 Social Activities: α =.84 Intimate Activities: α =.82
	I would like to use video-based technology in this context.	adapted from Davis, 1989	
	Video-based technology does not come into my house for this purpose.	adapted from Davis, 1989	
Perceived Benefits (5 Items)	Gain in safety	based on qualitative study	Overall: α =.85 Household Activities: α =.79 Social Activities: α =.79 Intimate Activities: α =.82
	Increased independence and autonomy	based on qualitative study	
	Faster reactions in emergencies	based on qualitative study	
	Gain in comfort and convenience	based on qualitative study	
	Relief for caring relatives	based on qualitative study	
Perceived Barriers (6 Items)	Invasion of Privacy	based on qualitative study	Overall: α =.82 Household Activities: α =.78 Social Activities: α =.78 Intimate Activities: α =.76
	Fear of Data Misuse	based on qualitative study	
	Sense of Surveillance	based on qualitative study	
	Fear of technical problems	based on qualitative study	
	Fear of false alarms	based on qualitative study	
	Feeling of incapacitation		
